# Differences in gut microbiota and its metabolic function among different fasting plasma glucose groups in Mongolian population of China

**DOI:** 10.1186/s12866-023-02852-7

**Published:** 2023-04-15

**Authors:** Yanchao Liu, Mingxiao Wang, Wuyuntana Li, Yumin Gao, Hailing Li, Ning Cao, Wenli Hao, Lingyan Zhao

**Affiliations:** 1 Department of Epidemiology, School of Public Health, Inner Mongolia Medical University, Inner Mongolia Autonomous Region, Hohhot, 010110 China; 2 Laboratory for Molecular Epidemiology in Chronic Diseases, Inner Mongolia Medical University, Inner Mongolia Autonomous Region, Hohhot, 010110 China; 3 Cspc Zhongqi Pharmaceutical Technology (Shijiazhuang) Co., Ltd, Shijiazhuang, Hebei Province 050035 China

**Keywords:** Type 2 diabetes mellitus, Metabolic function, Gut microbiota, Mongolian population, Carotene intake

## Abstract

**Background:**

Many studies reported the association between gut microbiota and type 2 diabetes mellitus (T2D), but it is still unclear which bacterial genus plays a key role and how the metabolic function of gut microbiota changes in the occurrence and development of T2D. Besides, there is a high diabetic prevalence in Mongolian population, which may be partly affected by their high calorie diet. This study identified the main bacterial genus influencing T2D in Mongolian population, and analyzed the changes of metabolic function of gut microbiome. The association between dietary factors and the relative abundance of main bacterial genus and its metabolic function was also studied.

**Methods:**

Dietary surveys and gut microbiota test were performed on 24 Mongolian volunteers that were divided into T2D (6 cases), PRET2D (6 cases) and Control group (12 cases) according to fasting plasma glucose (FPG) values. The relative abundance and metabolic function of gut microbiome from their fecal samples were measured by metagenomic analysis. Statistic method was used to evaluate the association between dietary factors and the relative abundance of the main bacterial genus or its metabolic function.

**Results:**

This study found that the *Clostridium* genus may be one of the key bacterial genera affecting the process of T2D. First, the relative abundance of *Clostridium* genus was significantly different among the three groups. Second, there was a higher relative abundance of metabolic enzymes of gut bacteria in PRET2D and T2D group than that in Control group. Third, a strong correlation between *Clostridium* genus and many metabolic enzymes was uncovered, many of which may be produced by the *Clostridium*. Last, carotene intake daily was negatively correlated with the *Clostridium* but positively correlated with tagaturonate reductase catalyzing interconversions of pentose and glucuronate.

**Conclusions:**

The gut *Clostridium* genus may play an important role in the development of T2D and it could be a potential biomarker for T2D in Mongolian population. Meanwhile, the metabolic function of gut bacteria has changed during the early stage of T2D and the changes in carbohydrate, amino acid, lipid or energy metabolism of *Clostridium* genus may play a critical role. In addition, the carotene intake may affect reproduction and metabolic function of *Clostridium* genus.

**Supplementary Information:**

The online version contains supplementary material available at 10.1186/s12866-023-02852-7.

## Background

Diabetes mellitus (DM) is one of the fastest-growing global health emergencies in the 21st century and 90% of that is type 2 diabetes mellitus (T2DM / T2D). In 2021, 537 million people estimated have diabetes and this number is projected to reach 643 million by 2030 [1]. China is a country with the largest number of diabetic patients, which brings a serious economic burden [1]. According a new survey, the T2D prevalence of Mongolian population in Inner Mongolia of China was 17.2%[2], that is a high rate comparing with the global average-level. Studies of the cause of T2D will be beneficial to the prevention and treatment of this disease. Growing evidence indicates gut microbiota plays an important role in development of T2D [3]. And the gut microbiome has changed before sugar regulation is impaired [4]. Comparing with healthy people, the patients with diabetes have a moderate disturbance in the intestinal bacteria and an increased number of pathogenic bacteria [3]. Similarly, PreT2D population has an abnormal intestinal flora [5]. This suggests that the gut microbiota changes dynamically during the occurrence and development of T2D. Therefore, we divided the population into T2D, preT2D and control group for comparative analysis. Dietary factors can affect the process of T2D by influencing gut microbiota [6]. A cohort study revealed that higher fruit intake-associated gut bacteria was associated with a lower risk of T2D [7]. Numerous evidences show that the change in dietary pattern can influence the gut microbial composition and diversity [8]. Meanwhile, some metabolites of gut microbiome are linked with the risk of T2D. And gut metabolites like short-chain fatty acids (SCFAs) yielded by fermentation of non-digestible carbohydrates in gut microbiome are mediators mediating communication between intestinal bacteria with host in T2D [9]. In this study, we concerned with how dietary factors and gut microbiota affect T2D. This study focused not only on the changes of the gut microbiota itself, but also on the changes of the metabolic function of that through metagenomic analysis. The method can truly reflect the composition and interaction of microbiota in a sample and be used to study the metabolic pathway and gene function at the molecular level [10–11]. Our data may contribute to promote the prevention and treatment of the T2D.

## Methods

### Participants

This study recruited Mongolian volunteers in Inner Mongolia of China, who met the following criteria. (1) Inclusion criteria: More than three generations of pure Mongolian; Aged 18 to 79 years; Meeting diagnostic criteria of T2D; New diagnosed cases of T2D and preT2D; The ratio of men to women is approximately 1: 1; No diarrhea in the recent week; Have not taken any antibiotics for nearly a month; No exposure to radioactive substances and radiation in recent three months; No gastrointestinal diseases; Voluntarily participate in the trial and sign the informed consent form. (2) Exclusion criteria: Other types of diabetes, such as type 1 diabetes, gestational diabetes and other special types of diabetes; Other types of endocrine disorders, such as primary aldosteronism, hyperthyroidism; Chronic infectious diseases such as chronic viral hepatitis and tuberculosis; Pregnant or lactating subjects; Subjects with mental diseases; Subjects with acute inflammation and trauma; Subjects with serious heart, brain, liver and kidney diseases, such as acute stroke, acute myocardial infarction and severe liver and kidney function damage; Subjects taking hypoglycemic drugs and other drugs for a long time.

### Dietary survey

We collected information of volunteers through face-to-face interviews. Food Frequency Questionnaire (FFQ) with 69-food items was used for the dietary survey and duration of the diet retrospect was one year.

### Fasting plasma glucose (FPG) test, physical examination and fecal samples collection

FPG test was completed in a hospital. We Measured the height, weight, waistline, hipline, systolic blood pressure (SBP) and diastolic blood pressure (DBP) of the participants used unified standard measurement tools. Waist to hip ratio (WHR) and body mass index (BMI) were calculated. Meanwhile, fecal samples of the participants were collected for the metagenomic analysis.

### Metagenomic analysis

#### 1. Sequencing results pretreatment

The total DNA extracted from fecal samples was constructed into a library and sequenced by Illumina PE150. Preprocessing the Raw Data obtained from the Illumina HiSeq sequencing platform using Readfq (V8, https://github.com/cjfields/readfq) was conducted to acquire the Clean Data for subsequent analysis. The specific processing steps were as follows: (1) removed the reads which contained low quality bases (default quality threshold value < 38) above a certain portion (default length of 40 bp); (2) removed the reads in which the N base had reached a certain percentage (default length of 10 bp); (3) removed reads which shared the overlap above a certain portion with Adapter (default length of 15 bp) [12, 13]. Considering the possibility of host contamination in the samples, Clean Data needed to be performed sequence alignment with the host database which by default uses Bowtie 2.2.4 software (Bowtie 2.2.4, http://bowtie-bio.sourceforge.net/bowtie2/index.shtml) to filter the reads that were of host origin, the parameters were as follows: --end-to-end, --sensitive, -I 200, -X 400 [13].

#### 2. Metagenome Assembly

(1) Single sample assembly.

The Clean Data was assembled and analyzed by SOAPdenovo software (V2.04, http://soap.genomics.org.cn/soapdenovo.html) [14], the parameters were set as follows: -d 1, -M 3, -R, -u, -F, -K 55 [15]. Then interrupted the assembled Scaftigs from N connection and leave the Scaftigs without N. All samples’ Clean Data was compared to each Scaffolds respectively by Bowtie2.2.4 software to acquire the PE reads not used and the parameters were: --end-to-end, --sensitive, -I 200, -X 400 [15].

(2) Mixed assembly.

All the reads not used in the forward step of all samples were combined and then used the software of SOAPdenovo (V2.04) for mixed assembly with the parameters same as single assembly. Break the mixed assembled Scaffolds from N connection and obtained the Scaftigs. Filtered the fragment shorter than 500 bp in all Scaftigs for statistical analysis both generated from single or mixed assembly.

#### 3. Gene prediction and abundance analysis

(1) The Scaftigs (> 500 bp) assembled from both single and mixed were all predicted the ORF by MetaGeneMark (V2.10, http://topaz.gatech.edu/GeneMark/) software, and filtered the length information shorter than 100nt from the predicted result with default parameters [16, 17].

(2) For ORF predicted, CD-HIT software (V4.5.8, http://www.bioinformatics.org/cd-hit) [18] was adopted to redundancy and obtain the unique initial gene catalogue (the genes here refers to the nucleotide sequences coded by unique and continuous genes), the parameters option were -c 0.95, -G 0, -aS 0.9, -g 1, -d 0 [19].

(3) The Clean Data of each sample was mapped to initial gene catalogue using Bowtie2.2.4 and got the number of reads to which genes mapped in each sample with the parameter setting were -end-to-end, --sensitive, -I 200, -X 400 [20]. Filtered the gene which the number of reads < 2 in each sample and obtained the gene catalogue (Unigenes) eventually used for subsequently analysis.

(4) Based on the number of mapped reads and the length of gene, counted the abundance information of each gene in each sample [21]. We calculated the abundance of any sample *S* as follow [22]:

Step 1: Calculation of the copy number of each gene:

$$ {b}_{i}=\frac{{x}_{i}}{{L}_{i}}$$

Step 2: Calculation of the relative abundance of gene i:

$$ {r}_{i}=\frac{{b}_{i}}{\sum _{j=1}^{n}\frac{{x}_{j}}{{L}_{j}}}$$

*b*_*i*_: the copy number of gene i in the sequenced data from samples *S*.

r_i_: the relative abundance of gene i in sample *S*.

x_i_: the number of mapped reads.

L_i_: the length of gene i.

(5) The basic information statistic, core-pan gene analysis, correlation analysis of samples and venn figure analysis of number of genes were all based on the abundance of each gene in each sample in gene catalogue.

#### 4. Taxonomy prediction

**(**1) DIAMOND [23] software (V0.9.9, https://github.com/bbuchfink/diamond/) was used to blast the Unigenes to the sequences of Bacteria, Fungi, Archaea and Viruses which are all extracted from the NR database (Version: 2018-01-02, https://www.ncbi.nlm.nih.gov/) of NCBI with the parameter setting are blastp, -e 1e-5. (2) For the finally aligned results of each sequence, as each sequence may have multiple aligned results, chose the result of which the e value < the smallest e value * 10 to take the LCA algorithm which was applied to system classification of MEGAN software to make sure the species annotation information of sequences [24].

(3) The table containing the number of genes and the abundance information of each sample in each taxonomy hierarchy (kingdom, phylum, class, order, family, genus, species) were obtained based on the LCA annotation result and the gene abundance table. The abundance of a specie in one sample was the sum of the gene abundance annotated for the specie. The gene number of a specie in a sample equaled the number of genes whose abundance are nonzero.

(4) The exhibition of generation situation of relative abundance, the exhibition of abundance cluster heat map, and NMDS (R vegan package, Version 2.15.3) decrease-dimension analysis were based on the abundance table of each taxonomic hierarchy.

(5) LEfSe analysis was used to look for the different species between groups [25]. First, a non-parametric factorial Kruskal-Wallis (KW) sum-rank test was used to test the species with significant differences in abundance between groups. Second, a group Wilcoxon rank sum test was used to determine the differences. And finally, linear discriminant analysis (LDA) was used to reduce dimensionality analysis and assess the impact size of significantly different species to the group by LDA Score. Permutation test between groups was used in Metastats analysis for each taxonomy and got the *p* value, then used Benjamini and Hochberg False Discovery Rate to correct *p* value and acquire *q* value. LEfSe analysis was conducted by LEfSe software (the default LDA score is 4).

#### 5. Common functional database annotations

(1) Adopted DIAMOND software (V0.9.9) to blast Unigenes to KEGG functional database (Version 2018-01-01, http://www.kegg.jp/kegg/) with the parameter setting of blastp, -e 1e-5 [20, 26]. For each sequence’s blast result, the best Blast Hit was used for subsequent analysis.

(2) Statistic of the relative abundance of different functional hierarchy, the relative abundance of each functional hierarchy equaled the sum of relative abundance annotated to that functional level.

(3) Based on the function annotation result and gene abundance table, the gene number table of each sample in each taxonomy hierarchy was obtained. The gene number of a function in a sample equaled the gene number that annotated to this function and the abundance was nonzero.

(4) Based on the abundance table of each taxonomy hierarchy, not only the counting of annotated gene numbers, the exhibition of the general relative abundance situation, the exhibition of abundance cluster heat map was conducted, but also comparative analysis of metabolic pathways between groups were performed.

### Statistical analysis

SPSS26 and R software were used for statistical analysis. The measurement data of normal distribution was expressed by mean ± standard deviation, which met the conditions of parameter test. The data were compared between groups by one-way ANOVA. The data that did not meet the conditions of parameter test were expressed by median (interquartile interval), and the comparison between groups was analyzed by rank sum test and multi classification logistic regression. Chi square test was used for counting data. Spearman nonparametric inertia analysis was used for correlation analysis. When *p < 0.05, **p < 0.01 and ***p < 0.001, the differences were statistically significant. Some figures were completed using the Wekemo Bioincloud (https://www.bioincloud.tech).

## Results

### Characteristics of study participants

24 Mongolian participants were screened out from 160 volunteers by the inclusion and exclusion criteria. They were divided into three groups, diabetes group (T2D, 6 cases), prediabetes group (PRET2D, 6 cases) and normal glucose control group (Control, 12 cases), according to FPG values. Every T2D patient was matched a PRET2D case and two control numbers with same gender and similar age. The detailed information of each participant was showed in supplemental Table 1. Excepting FPG, waistline and WHR, there were no statistical differences of other characteristics between the three groups, including age, BMI, hipline, SBP and DBP (Table 1).

**Table 1:** Characteristics of participants in the three groups

Characteristics	T2D (n = 6)	PRET2D (n = 6)	Control (n = 12)	*F* value	*p* value
FPG, mmol/L	10.81 ± 5.54	6.38 ± 0.29	5.57 ± 0.27	7.74	0.03*
Age, years	61.33 ± 7.71	59.17 ± 8.47	56.25 ± 8.31	0.82	0.46
BMI	27.41 ± 2.52	26.84 ± 1.35	24.89 ± 3.40	1.94	0.17
Waistline, cm	101.33 ± 6.83	95.83 ± 6.21	89.00 ± 7.83	6.13	0.08*
Hipline, cm	104.17 ± 6.11	102.25 ± 5.60	98.91 ± 6.61	1.55	0.24
WHR	0.97 ± 0.03	0.94 ± 0.08	0.90 ± 0.05	3.76	0.04*
SBP, mmHg	132.33 ± 17.13	134.83 ± 15.70	129.75 ± 16.88	0.19	0.83
DBP, mmHg	82.17 ± 12.37	88.50 ± 6.38	87.25 ± 10.31	0.70	0.51

* means a difference between three groups is statistically significant.

## Metagenomic analysis

### 1. Species analysis

#### (1) Number of genes

**Fig. 1:**
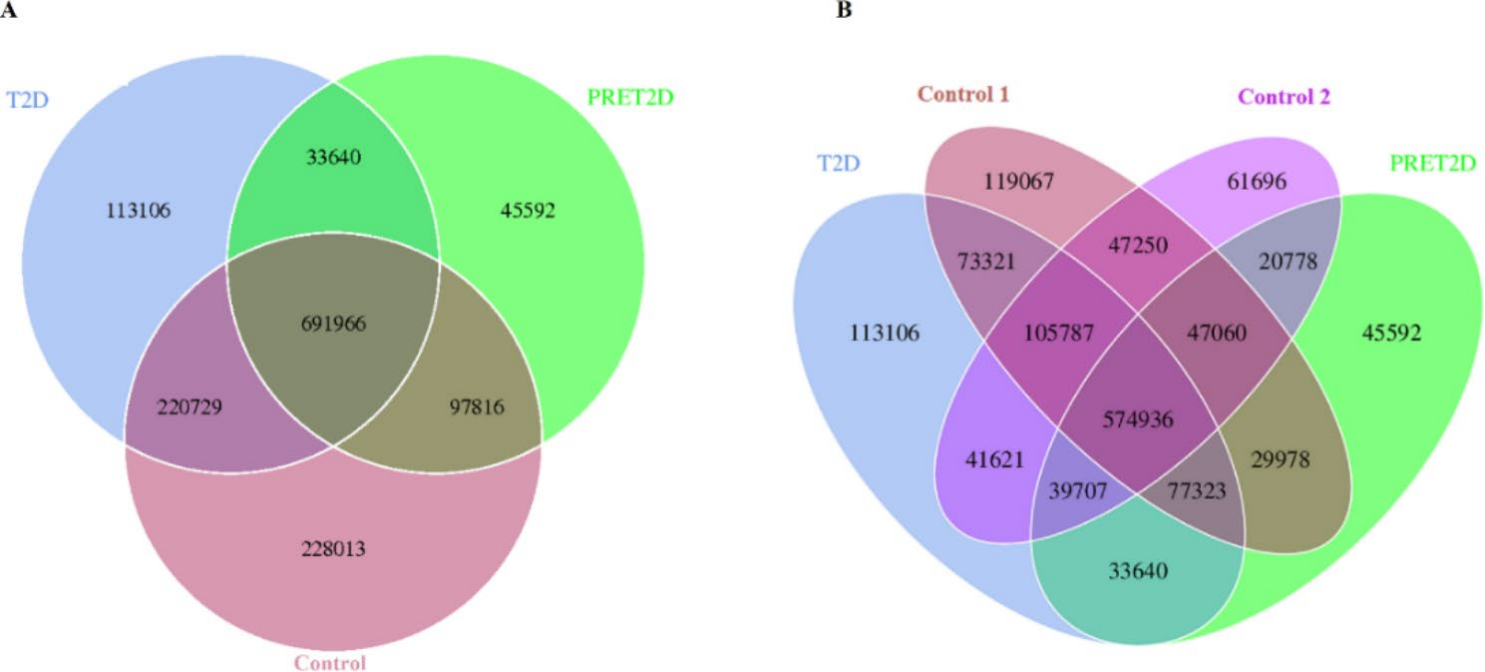
Common and unique characteristic of genes among the different groups. Overlapping regions represent common part, other regions mean unique part, numbers are gene quantity. **A** A flower map showing difference between T2D, PRET2D and Control groups; **B** A Venn map showing difference between T2D, PRET2D, Control 1 and Control 2 groups

Number of genes in T2D, PRET2D and Control group was counted respectively. Comparing the gene sequences between the three groups, we sorted out common and unique genes, which was showed by a flower map (Fig. 1A). From the figure we can see clearly that Control group owned the largest quantities of unique genes with 228,013. Second came T2D group with 113,106. Following the T2D came PRET2D with 45,592, that was the smallest. It is interesting to note that the total genes of the three groups had the similar trend with that of above, Control (1,238,524) > T2D (1,059,495) > PRET2D (869,014). In addition, to study changes of gene number in different groups with different FPG value in a healthy state, Control group was divided into Control 1 with low value (5.4 ± 0.2 mmol/L) and Control 2 with high value (5.8 ± 0.1 mmol/L). Then the difference of the four groups was shown by a Venn map (Fig. 1B). It is noticeable that there was an obvious disagree between Control 1 and Control 2, which the unique genes in Control 1 were almost twice as many as that in Control 2. That means, even in the normal group, the genes of gut microbiota were variant in different FPG level. We could conclude that following the FPG value rising, the number of specific genes decreased, but that increased again at T2D state. These difference of genes in different groups could be reflected the result of changes in the number and function of microbiome.

#### (2) Analysis of the relative abundance of microbial species

**Fig. 2:**
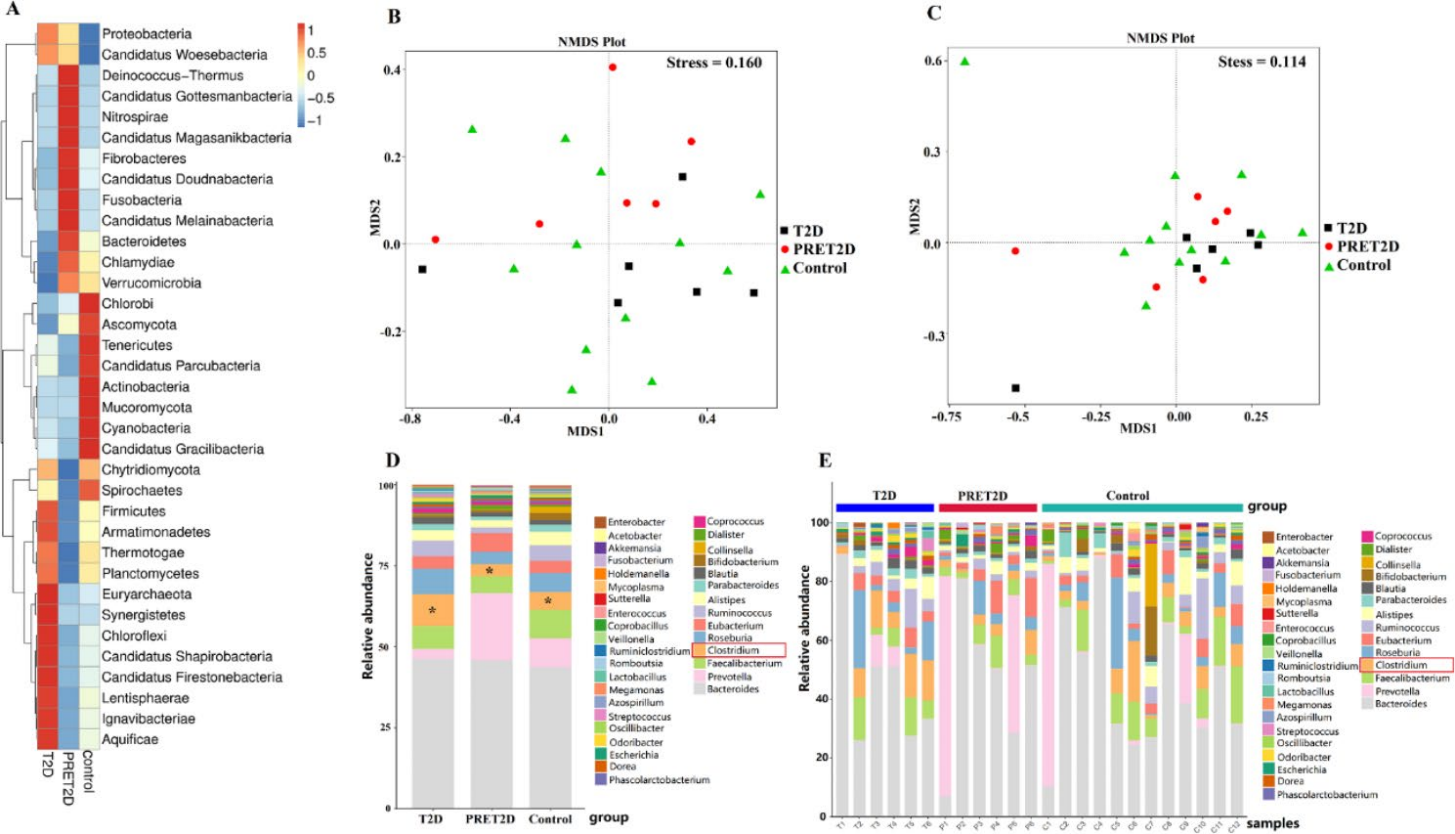
Results of gut microbiome abundance analysis in phylum and genus hierarchies. **A** Cluster heat map of abundance in phylum in the three groups. A different profile between the groups without statistic difference. The redder the color, the more quantity, while the bluer, the less quantity. **B** NMDS decrease-dimension analysis in phylum in the three groups with no significant difference. **C** NMDS decrease-dimension analysis in genus in the three groups with no significant difference. **D** Abundance comparison in genus. The abundance of *Clostridium* differed significantly between the three groups, *p < 0.05. **E** Abundance comparison of each sample in genus

Cluster heat map and NMDS decrease-dimension analysis were used to compare diversity difference between groups. There was not a significant difference between each level of gut microbiome and we showed partly results in Fig. 1, including a heat map of phylum, two NMDS maps of phylum and genus. The top 35 species with the highest relative abundance (top 35) were statistically analyzed respectively by ANOVA. We found that there was significant difference among the three groups in a family level and genus level, which was *Clostridiaceae* family(*k_Bacteria; p_Firmicutes; c_Clostridia; o_Clostridiales; f_Clostridiaceae*)(*p* = 0.047) and *Clostridium* genus (*k_Bacteria; p_Firmicutes; c_Clostridia; o_Clostridiales; f_Clostridiaceae; g_Clostridium*) (*p* = 0.049) (Fig. 2). The number of *Clostridiaceae* family and *Clostridium* genus all decreased in PRET2D group, but increased in T2D group with the most number, this difference was similar to the result of unique genes above.

#### (3) LEfSe analysis of different species among groups

**Fig. 3:**
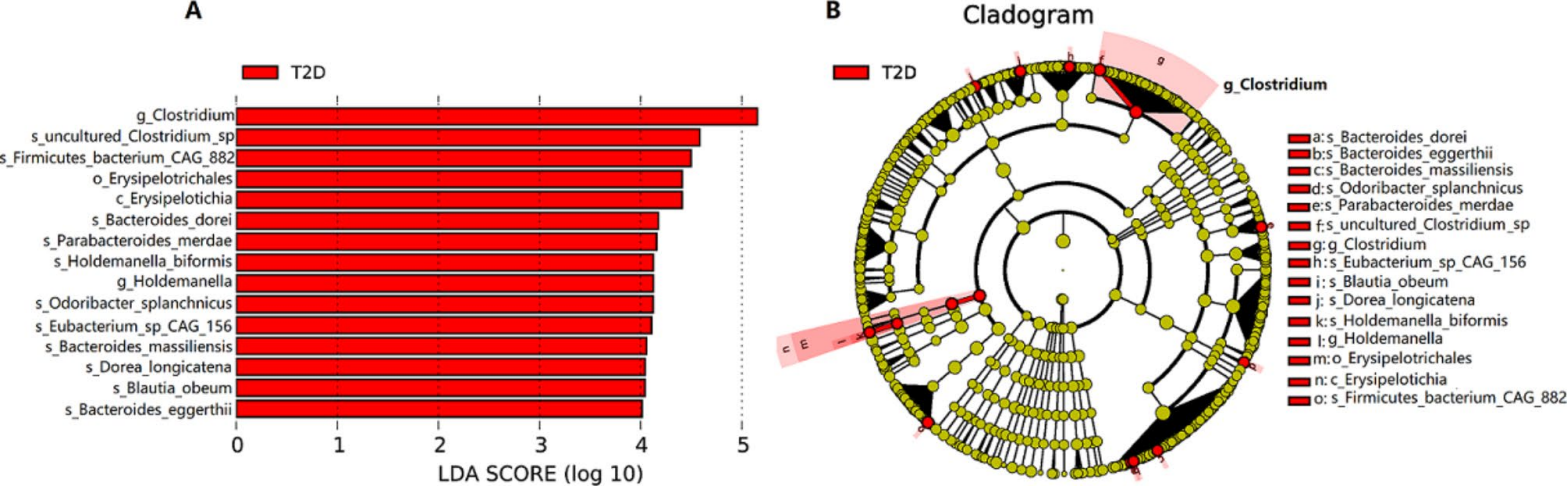
Results of the LEfSe analysis. **A** A distribution diagram of LDA score of different species. Length of histogram represents an influence of different species. Species with LDA score is higher than 4 can be regarded as biomarkers with statistical difference among groups. **B** A evolutionary branch diagram of different species. Circles radiating from inside to outside represent the classification hierarchy from phylum to species. Each small circle in different classification hierarchies represents a classification at this hierarchy. The diameter of the small circle is directly proportional to the relative abundance. Species with no significant difference are uniformly colored as yellow, whereas the species with significant difference which can be used as a biomarker for T2D are colored as red. The red node indicates the microbial flora that play an important role in the red group

In order to screen bacterial biomarkers with significant difference among groups, we measured different species among groups by LEfSe analysis. The criteria was that it had significantly impact to the group when the LDA Score over 4. The analysis showed that the *Clostridium* genus has the highest LDA score in T2D group, whereas there was no species whose LDA score over 4 in PRET2D and Control. As a result, it could be used as a potential bacterial biomarker for T2D (Fig. 3).

### 2. Analysis of function

#### (1) Statistics on the number of genes annotated in the KEGG database

**Fig. 4:**
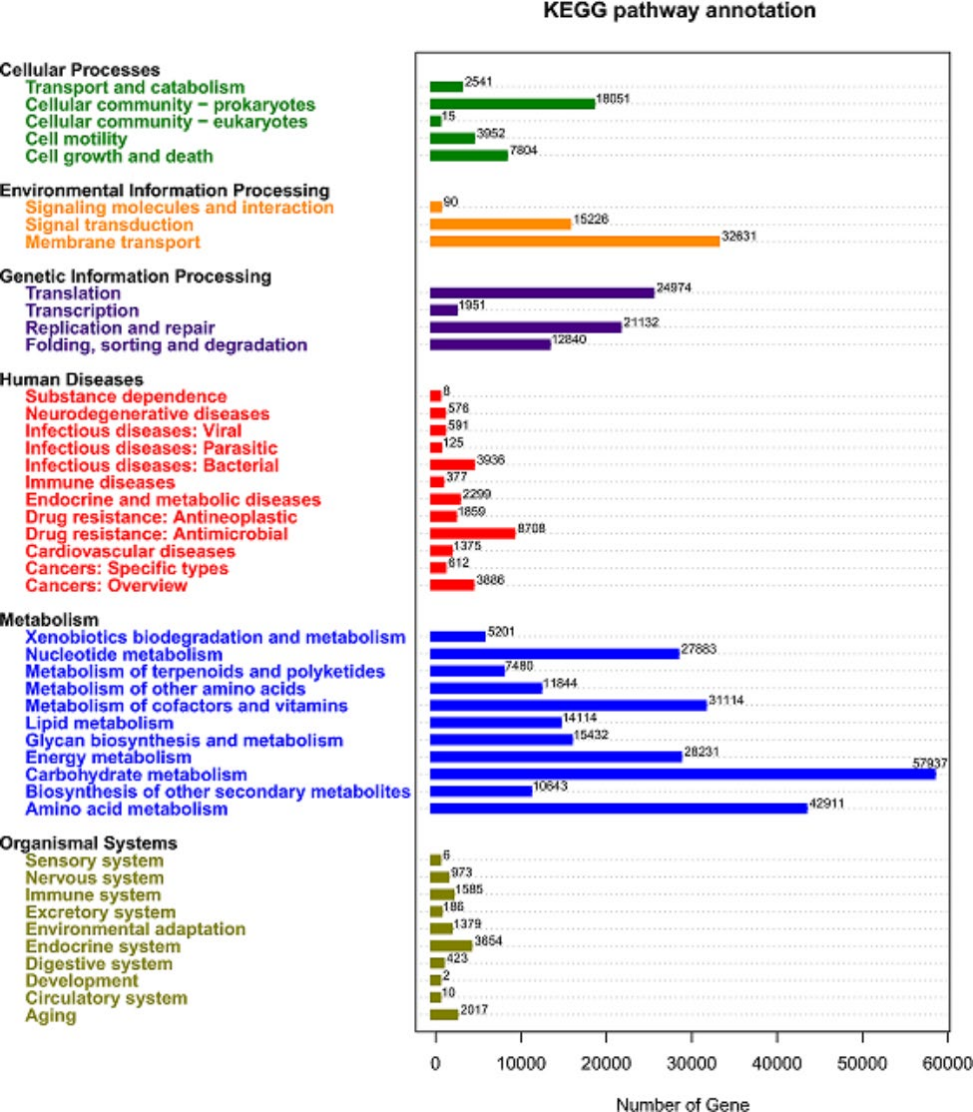
Number of genes that were annotated in the first level in KEGG database was dispaled with different colors. Most of which were metabolism related genes in blue and the highest is carbohydrate metabolism

Unigenes were blasted in the KEGG database. Most of the genes annotated in the first level of KEGG database were metabolism related genes, especially genes of carbohydrate metabolism were the highest with 57,937 (Fig. 4). In this study, we focused on the difference of the metabolic function of gut microbiotic and the the difference of carbohydrate metabolism of *Clostridium* was priority, among T2D, PRET2D and Control groups.

#### (2) Functional analysis

**Fig. 5:**
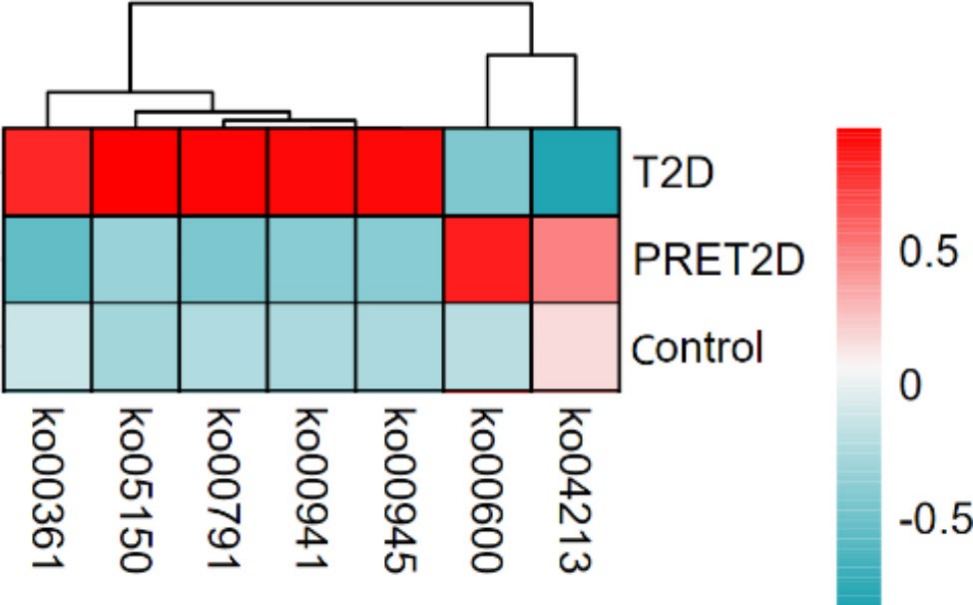
A result of difference of relative abundance of functionnal genes between three groups in level 3. Five kinds of functional genes were the highest in T2D and two ones were the highest in PRET2D than that in other groups. The redder the color, the more quantity, while the bluer, the less quantity

A camparation of relative abundance of functional genes was carryed out among the three groups in level 1,level 2 and level 3 of KEGG database by ANOVA. There was no significant diffrence in level 1, while digestive system in level 2 belonging to organismal systems was significantly diffrent among groups (PRET2D > Control > T2D, *p* = 0.034). That implyed the digestive function of gut microbiota had a trend that it would become more activity in preT2D stage then decline the lowest level when the T2D occurred. Seven significant differences in level 3 were observed, including five of metabolic function (ko00600, ko00361, ko00791, ko00941 and ko00945) (Table 2). The trend of difference of ko00600 among the three groups was consistent with that of digestive system, while other four metabolic functions were all the highest in T2D group (Fig. 5).

**Table 2:** Seven functional genes with significant difference in level 3, annotation and *p* value

Name	Level1	Level 2	Level 3	*p* value
ko00600	Metabolism	Lipid metabolism	Sphingolipid metabolism	0.045
ko00361	Metabolism	Xenobiotics biodegradation and metabolism;	Chlorocyclohexane and chlorobenzene degradation	0.043
ko00791	Metabolism	Xenobiotics biodegradation and metabolism	Atrazine degradation	0.022
ko00941	Metabolism	Biosynthesis of other secondary metabolites	Flavonoid biosynthesis	0.026
ko00945	Metabolism	Biosynthesis of other secondary metabolites;	Stilbenoid, diarylheptanoid and gingerol biosynthesis	0.026
ko04213	Organismal Systems;	Aging	Longevity regulating pathway - multiple species	0.046
ko05150	Human Diseases	Infectious diseases: Bacterial	Staphylococcus aureus infection	0.019

#### (3) Difference analysis of the relative abundance of metabolic enzymes

**Fig. 6:**
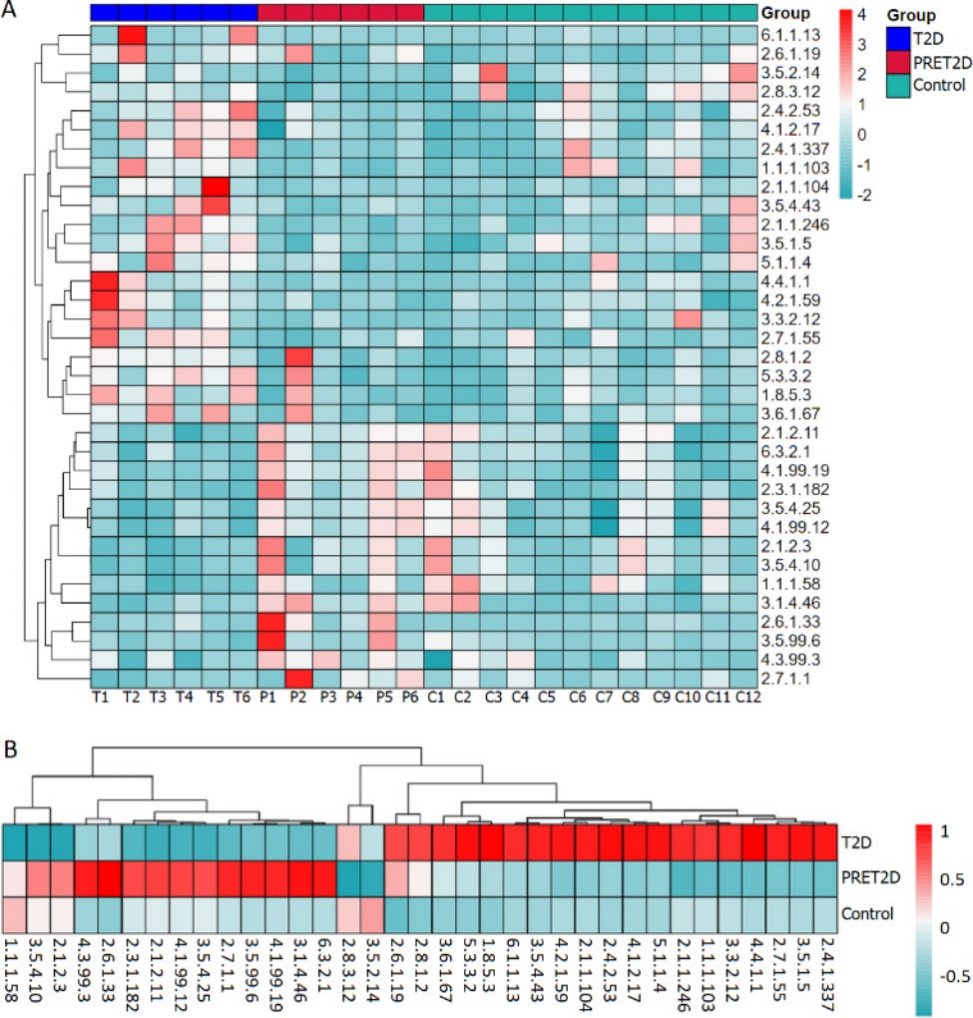
Relative abundances of 35 metabolic enzymes with significant difference between T2D, PRET2D and Control group in gut microbiota were showed. **A** A clustering heat map of the 35 metabolic enzymes in each sample. **B** A clustering heat map of the 35 metabolic enzymes in the three groups. The redder the color, the more quantity, while the bluer, the less quantity

Many genes of metabolic enzymes were discovered by the annotated function of KEGG. 35 of them were identified significant difference in relative abundance among the 3 groups by ANOVA. It is interesting to note that most of that were involved in carbohydrate metabolism, amino acid metabolism and metabolism of cofactors and vitamins. Besides, some of them participated in multiple metabolic pathways (Table 3). The number of most enzymes were the highest in T2D or PRET2D group, while the quantity of tagaturonate reductase catalyzing the interconversions of pentose and glucuronate (1.1.1.58) gradually declined from Control to PRET2D to T2D group. Therefore, a hypothesis was estimated that the metabolic function of gut microbiota changed significantly in the T2D stage and even in the early stage of T2D (Fig. 6).

**Table 3:** Affiliation, Name, Function and *p* value of the 35 gut microbiotic enzymes with significant difference between T2D, PRET2D and Control group

Level 2	EC ID	Name	Function	*p* value
Carbohydrate metabolism	1.1.1.58	tagaturonate reductase	pentose and glucuronate interconversions.	0.049
	2.3.1.182	Transferases	C5-Branched dibasic acid metabolism; Valine, leucine and isoleucine biosynthesis.	0.047
	2.4.2.53	undecaprenyl-phosphate 4-deoxy-4-formamido-L-arabinose transferase	Amino sugar and nucleotide sugar metabolism.	0.015
	2.6.1.19	4-aminobutyrate-2-oxoglutarate transaminase	Butanoate and Propanoate metabolism; Alanine, aspartate, glutamate, beta-Alanine metabolism.	0.015
	2.7.1.1	Hexokinase	Starch, Sucrose, Glycolysis, Gluconeogenesis, Galactose, Fructose and mannose, Amino sugar and nucleotide sugar metabolism; Streptomycin, Neomycin, kanamycin, gentamicin biosynthesis.	0.022
	2.7.1.55	allose kinase	Fructose and mannose metabolism.	0.027
	2.8.3.12	glutaconate CoA-transferase	Butanoate metabolism; Styrene degradation.	
	3.5.99.6	glucosamine-6-phosphate deaminase	Amino sugar and nucleotide sugar metabolism.	0.027
	4.1.2.17	L-fuculose-phosphate aldolase	Fructose and mannose metabolism.	0.017
Amino acid metabolism	1.1.1.103	L-threonine 3-dehydrogenase	Glycine, serine and threonine metabolism.	
	2.1.1.104	caffeoyl-CoA O-methyltransferase	Phenylalanine metabolism; Flavonoid biosynthesis; Phenylpropanoid biosynthesis; Stilbenoid, diarylheptanoid, gingerol biosynthesis.	0.026
	2.8.1.2	3-mercaptopyruvate sulfurtransferase	Cysteine and methionine metabolism.	0.045
	3.3.2.12	oxepin-CoA hydrolase	Phenylalanine metabolism	0.033
	3.5.1.5	Urease	Arginine biosynthesis; Nucleotide metabolism; Purine metabolism; Atrazine degradation.	0.017
	3.5.2.14	N-methylhydantoinase (ATP-hydrolysing)	Arginine and proline metabolism.	0.045
	4.4.1.1	cystathionine gamma-lyase	Cysteine and methionine metabolism; Glycine, serine and threonine metabolism; Selenocompound metabolism.	0.007
	5.1.1.4	proline racemase	Arginine and proline metabolism	0.020
	6.1.1.13	D-alanine-poly(phosphoribitol) ligase	D-Alanine metabolism.	0.042
	6.3.2.1	pantoate-beta-alanine ligase (AMP-forming)	beta-Alanine metabolism; Pantothenate and CoA biosynthesis.	0.018
Metabolism of cofactors and vitamins	2.1.2.3	phosphoribosylaminoimidazolecarboxamide formyltransferase	One carbon pool by folate.	0.046
	2.1.2.11	3-methyl-2-oxobutanoate hydroxymethyltransferase	Pantothenate and CoA biosynthesis.	0.026
	3.5.4.25	GTP cyclohydrolase II	Riboflavin metabolism; Folate biosynthesis;	0.038
	3.6.1.67	dihydroneopterin triphosphate diphosphatase	Folate biosynthesis.	0.043
	4.1.99.12	3,4-dihydroxy-2-butanone-4-phosphate synthase	Riboflavin metabolism.	0.036
	4.1.99.19	2-iminoacetate synthase	Thiamine metabolism.	0.030
	4.3.99.3	7-carboxy-7-deazaguanine synthase	Folate biosynthesis.	0.027
Lipid metabolism	2.4.1.337	1,2-diacylglycerol 3-alpha-glucosyltransferase	Glycerolipid metabolism.	0.017
	3.1.4.46	glycerophosphodiester phosphodiesterase	Glycerophospholipid metabolism.	0.013
	4.2.1.59	3-hydroxyacyl-[acyl-carrier-protein] dehydratase	Fatty acid biosynthesis.	0.020
Nucleotide metabolism	3.5.4.10	IMP cyclohydrolase	Purine metabolism.	0.047
Energy metabolism	1.8.5.3	respiratory dimethylsulfoxide reductase	Sulfur metabolism.	0.011
	2.1.1.246	[methyl-Co (III) methanol-specific corrinoid protein]-coenzyme M methyltransferase	Methane metabolism.	0.023
Metabolism of terpenoids and polyketides	2.6.1.33	dTDP-4-amino-4,6-dideoxy-D-glucose transaminase	Polyketide sugar unit biosynthesis; Biosynthesis of other secondary metabolites;Acarbose and validamycin biosynthesis.	0.007
	5.3.3.2	sopentenyl-diphosphate Delta-isomerase	Terpenoid backbone biosynthesis.	0.013
Xenobiotics metabolism	3.5.4.43	hydroxydechloroatrazine ethylaminohydrolase	Atrazine degradation.	0.032

### 3. Correlation analysis of dietary factors, *Clostridium* genus and metabolic enzymes

#### (1) Correlation analysis of dietary factors and *Clostridium* genus

**Fig. 7:**
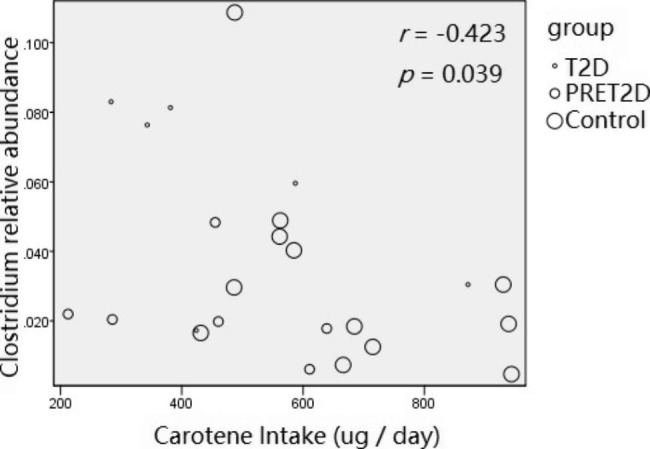
Negative correlation between the carotene intake daily and the relative abundance of *Clostridium* genus. The biggest bubbles were samples from the Control and the medium ones were from the PERT2D, the smallest ones were from the T2D.

We calculated the average daily intake of food or nutrients for each participant. The difference of carotene intake in 3 groups was statistically significant by ANOVA (481.3 ± 216.9 µg vs. 444.0 ± 170.3 µg vs. 666.0 ± 183.8 µg) (*p* = 0.048). Meanwhile, the carotene intake and seafood intake showed a negative correlation with FPG (spearman, *r* = -0.503, *p* = 0.012; *r* = -0.570, *p* = 0.004). In addition, the carotene intake and the relative abundance of *Clostridium* genus was negatively correlated (spearman, *r* = -0.423, *p* = 0.039) (Fig. 7).

#### (2) Correlation analysis of dietary factors, *Clostridium* genus and metabolic enzymes

**Fig. 8:**
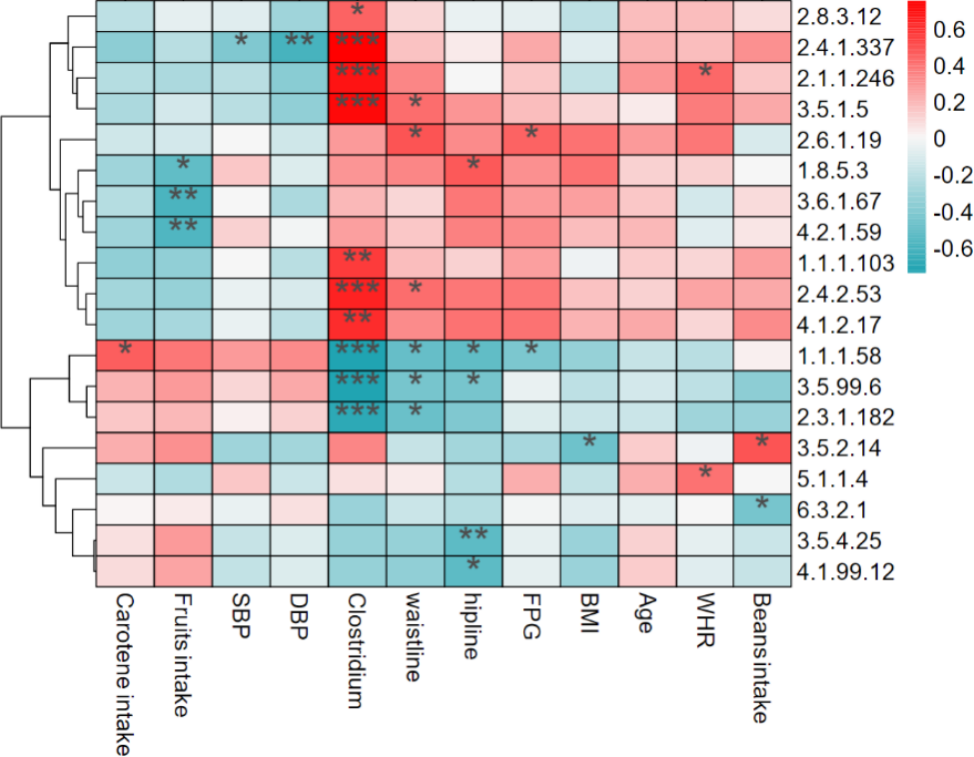
Correlations of 19 metabolic enzymes with *Clostridium* genus and other factors. The redder the color, the more quantity, while the bluer, the less quantity. *p < 0.05, **p < 0.01 and ***p < 0.001

We found 19 in the 35 metabolic enzymes above could be produced by *Clostridium* genus through analyzing the result of KEGG annotating. We counted correlations of the relative abundance of the19 metabolic enzymes with *Clostridium* genus and other factors that may be relevant, including age, FPG, BMI, waistline, hipline, WHR, DBP, SBP, carotene intake, beans intake and fruits intake (Fig. 8). There were ten metabolic enzymes significantly correlated to *Clostridium* genus, among which three have negative and seven have positive association. The three enzymes were tagaturonate reductase (1.1.1.58), glucosamine-6-phosphate deaminase (3.5.99.6) and transferases (2.3.1.182) respectively, which were all involved in carbohydrate metabolism. The seven enzymes were glutaconate CoA-transferase (2.8.3.12), 1,2-diacylglycerol 3-alpha-glucosyltransferase (2.4.1.337), [methyl-Co(III) methanol-specific corrinoid protein]-coenzyme M methyltransferase (2.1.1.246), Urease (3.5.1.5), L-threonine 3-dehydrogenase (1.1.1.103), undecaprenyl-phosphate 4-deoxy-4-formamido-L-arabinose transferase (2.4.2.53) and L-fuculose-phosphate aldolase (4.1.2.17 ) respectively, some of the them involved in carbohydrate metabolism, amino acid metabolism, some participated in lipid metabolism and energy metabolism. Carotene intake was positively related with tagaturonate reductase (1.1.1.58) that catalyzes the interconversions of pentose and glucuronate. Fruits intake showed a negative relation with respiratory dimethylsulfoxide reductase (1.8.5.3), dihydroneopterin triphosphate diphosphatase (3.6.1.67) and 3-hydroxyacyl-[acyl-carrier-protein] dehydratase (4.2.1.59), that participates in the sulfur metabolism, folate biosynthesis and fatty acid biosynthesis respectively. Beans intake was related to amino acid metabolic enzymes (3.5.2.14, 6.3.2.1). Blood pressure is negatively correlated with glycerolipid metabolic enzymes (2.4.1.337). Waistline, hipline and BMI also had correlation with metabolic enzymes.

## Discussion

In the present study of small population, we uncovered a difference in gut bacterial genus level between different T2D conditions. It means that we may have chance to screen out some specific species or strains with critical roles to regulate process of T2D from the *Clostridium* genus. It is meaningful because lots evidence has highlighted the importance of individual species or strains of the microbiome in human health [27]. In the Mongolian population, the *Clostridium* genus was declined in PRET2D group compared to Control group, that was agreed with another study in Danish adults [28]. However, in the T2D group, the genus was the highest than other two groups, that was a different result contrasting that of another research in ethnic Han population, which the genus’ quantity in T2D was lower than that in control [29]. Meanwhile, similar results as the study were not discovered in other researches [4, 30, 31], that could be contributed to the difference of testing method. We also found a difference in species level, *Firmicutes bacterium* CAG:341. There was a graduate increasing trend of the strain from Control to PRET2D to T2D. However, it is an unclassified species in Firmicutes and did not have significant correlation with the main changed metabolic enzymes in the study, we did not analyze that overmuch for keeping the story compactness. Fortunately, a team has constructed a Mongolian Gut Genome catalogue, comprising 802 closed and 5,927 high-quality metagenome-assembled genomes, providing a high-quality, large-scale resource for studying gut flora of Mongolian population [32]. That will be helpful to demonstrate the crucial intestinal bacteria in affecting the development of T2D in Mongolian.

We selected the *Clostridium* genus as a potential bacterial biomarker for T2D, apart from it was the largest difference genus in T2D, it had significant statistic difference between groups in the study, whereas other species or genera did not have the difference. Besides, as a diagnostic biomarker, it should be easy to test, so we can test the genus by qPCR in a normal laboratory with qPCR machine. However, the other species need more complicated technique like sequencing to identified. Meanwhile, *Clostridium* genus contains many butyrate-producing bacteria that were known as a friendly group for human health [9, 33, 34]. Although the quantity of the genus was higher in T2D, that did not mean that of butyrate-producing bacteria also rose. We may have a chance to isolate some crucial species that are involved in butyrate metabolic in the genus to be treatment biomarkers in future.

Besides, changes of metabolic function in gut microbiome were significantly related to T2D. Especially the changes of the metabolic pathway of carbohydrate played an important role, which was consistent with other studies [35]. Increasing evidence described that gut microbiota regulate sugar metabolism of the host by gut metabolites like SCFAs mediating, that were proved good for regulating T2D [9]. There were 9 metabolic enzymes significantly correlating with *Clostridium* in relative abundance. What the most interesting was the enzymes that negatively associating to the genus were all in carbohydrate metabolic pathways. It means that we should pay attention to mechanisms of effecting process of T2D in carbohydrate metabolic pathways of gut *Clostridium* genus. Meanwhile, the carotene intake may affect the reproduction and metabolic function of *Clostridium* genus.

Admittedly, there are several limitations in the present study. First, although the T2D patients and PreT2D participants were all new diagnosis, excluding the interference of drug treatment and other diseases, the sample size was small, so there would be sampling errors. Second, the dietary assessment was based on FFQ, which was subject to recall bias and measurement error. What’ s more, we could not know the methods of cooking that affect intake of nutrients by body. Consequently, the results need to be further confirmed by more population experiments.

## Conclusions

There was a significant difference in the relative abundance and metabolic function of gut microbiome among T2D, PRET2D and Control groups. And *Clostridium* genus can be a potential biomarker for T2D in Mongolian population. Meanwhile, compared with Control, PRT2D and T2D had an increased metabolic function of gut microbiota. The changes of the metabolic function of carbohydrate, amino acid, lipid and energy of *Clostridium* may play an important role. In addition, the carotene intake may negatively regulate the *Clostridium* but positively regulate tagaturonate reductase of the genus.

## Electronic supplementary material

Below is the link to the electronic supplementary material.

Supplementary Material 1

Supplementary Material 2

## Data Availability

The detail data and materials available please see http://www.ebi.ac.uk/arrayexpress/help/FAQ.html#cite.
